# Stress testing *Λ*CDM with high-redshift galaxy candidates

**DOI:** 10.1038/s41550-023-01937-7

**Published:** 2023-04-13

**Authors:** Michael Boylan-Kolchin

**Affiliations:** Department of Astronomy, The University of Texas at Austin, Austin, TX USA

**Keywords:** Cosmology, Galaxies and clusters

## Abstract

Early data from the James Webb Space Telescope (JWST) have revealed a bevy of high-redshift galaxy candidates with unexpectedly high stellar masses. An immediate concern is the consistency of these candidates with galaxy formation in the standard *Λ*CDM cosmological model, wherein the stellar mass (*M*_⋆_) of a galaxy is limited by the available baryonic reservoir of its host dark matter halo. The mass function of dark matter haloes therefore imposes an absolute upper limit on the number density *n* (>*M*_⋆_, *z*) and stellar mass density *ρ*_⋆_ (>*M*_⋆_, *z*) of galaxies more massive than *M*_⋆_ at any epoch *z*. Here I show that the most massive galaxy candidates in JWST observations at *z* ≈ 7–10 lie at the very edge of these limits, indicating an important unresolved issue with the properties of galaxies derived from the observations, how galaxies form at early times in *Λ*CDM or within this standard cosmology itself.

## Abstract (web summary)

Early James Webb Space Telescope (JWST) results suggest a high level of star formation in the first 500 million years of the Universe. A study of the available mass from dark matter haloes shows that unexpectedly high-mass JWST galaxy candidates may challenge the prevailing cosmological model.

## Main

*Λ* cold dark matter model (*Λ*CDM)-like cosmological models share a similar basic assumption: baryons and dark matter are well mixed at very early times, and as baryons collapse into dark matter haloes, the maximum amount of baryonic material within a halo will be equal to *M*_b_ = *f*_b_ *M*_halo_, where *f*_b_ ≡ *Ω*_b_/*Ω*_m_ is the cosmic baryon fraction. This, in turn, bounds the total stellar content of a dark matter halo: *M*_⋆_(*M*_halo_) ≤ *M*_b_(*M*_halo_). I show how this simple relation can be used as a stringent test of either cosmological models with minimal assumptions about galaxy formation, or the reliability of photometric selection and physical characterization of high-redshift galaxy candidates. My analysis is in many ways similar to that of Behroozi and Silk^1^, who connected cumulative number densities of dark matter haloes to high-redshift galaxy stellar mass functions (see also Steinhardt et al.^2^), although I also consider the maximal cumulative stellar mass density allowed in *Λ*CDM. The question of the consistency of stellar mass functions and the underlying cosmological dark matter halo mass functions has become considerably more urgent with the release of the first data from the James Webb Space Telescope (JWST), and with it, a swarm of high-redshift galaxy candidates^3–11^.

## Assumptions

I adopt the base *Λ*CDM model of the Planck Collaboration^12^, which assumes no spatial curvature and initial conditions that are Gaussian and adiabatic, as the standard cosmological model. I use best-fit values for cosmological parameters based on the plik TT,TE,EE + lowE + lensing likelihood applied to the full mission data. The relevant parameters and values for this work are the present-day Hubble constant, *H*_0_ = 67.32 km s^−1^ Mpc^−1^; the *z* = 0 density parameter for matter, *Ω*_m_ = 0.3158 (which includes baryons, dark matter and non-relativistic neutrinos); the slope of the primordial power spectrum of density fluctuations, *n*_s_ = 0.96605; the root mean square (r.m.s.) amplitude of the linear matter power spectrum at *z* = 0 as measured in spheres of radius 8 h^−1^ Mpc, *σ*_8_ = 0.8120; and the cosmic baryon fraction, *f*_b_ ≡ *Ω*_b_/*Ω*_m_ = 0.156 (ref. ^12^).

With these values, the linear matter power spectrum is specified at all times relevant for structure formation. The non-linear density field, home to the dark matter haloes that host galaxies, must be computed numerically. However, a long line of research starting with Press and Schechter^13^ has been devoted to connecting the abundance of dark matter haloes as a function of redshift and mass to the underlying linear matter power spectrum. In what follows, I use the Sheth and Tormen^14^ dark matter halo mass function d*n*(*M*, *z*)/d*M*—the number of dark matter haloes of mass *M* per unit mass per unit co-moving volume at redshift *z*—to compute the co-moving number density of haloes above a given halo mass threshold,

1 $$n( > {M}_{{{{\rm{halo}}}}},z)=\int\nolimits_{{M}_{{{{\rm{halo}}}}}}^{\infty }{\mathrm{d}}M\frac{{\mathrm{d}}n(M,z)}{{\mathrm{d}}M}$$

and the co-moving mass density in haloes more massive than *M*_halo_,

2 $${\rho }_{{{{\rm{m}}}}}( > {M}_{{{{\rm{halo}}}}},z)=\int\nolimits_{{M}_{{{{\rm{halo}}}}}}^{\infty }{\mathrm{d}}M\,M\frac{{\mathrm{d}}n(M,z)}{{\mathrm{d}}M}.$$

These translate directly to upper limits on the statistics of galaxies through the straightforward assumption that the largest stellar content a halo can have, given its cosmic allotment of baryons, is ${M}_{\star ,\max }={f}_{{{{\rm{b}}}}}\,{M}_{{{{\rm{halo}}}}}$. More generally, we may write *M*_⋆_ = *ϵf*_b_*M*_halo_, with *ϵ* ≤ 1.0 being the efficiency of converting baryons into stars.

The cumulative co-moving number density of dark matter haloes more massive than *M*_halo_ thus sets an upper limit on the co-moving number density of galaxies more massive than *M*_⋆_,

3 $${n}_{{{{\rm{gal}}}}}( > {M}_{\star })\le {n}_{{{{\rm{halo}}}}}( > {M}_{\star }/{f}_{{{{\rm{b}}}}}).$$

Similarly, the cumulative co-moving density of collapsed mass sets an upper limit on the density of collapsed baryons, *ρ*_b_(>*M*_halo_) = *f*_b_ *ρ*_m_(>*M*_halo_), which in turn strictly bounds the co-moving mass density of stars contained in haloes more massive than *M*_halo_,

4 $${\rho }_{\star }( > {M}_{{{{\rm{halo}}}}})\le {f}_{{{{\rm{b}}}}}\,{\rho }_{{{{\rm{m}}}}}( > {M}_{{{{\rm{halo}}}}}),$$

and the density of stars contained in galaxies above a given *M*_⋆_,

5 $${\rho }_{\star }( > {M}_{\star })\le {f}_{{{{\rm{b}}}}}\,{\rho }_{{{{\rm{m}}}}}( > {M}_{\star }/{f}_{{{{\rm{b}}}}}).$$

## Results

**Fig. 1:**
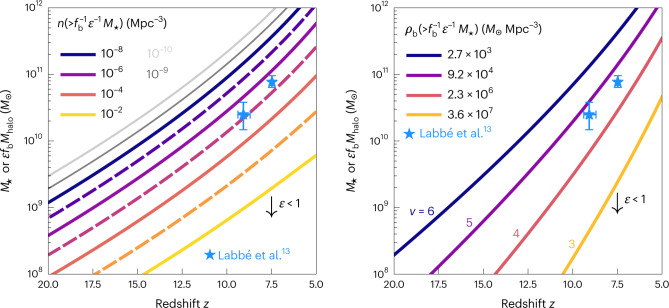
Limits on the abundance of galaxies as a function of redshift. Curves show the relationship between *M*_⋆_ and *z* at fixed cumulative halo abundance (left) and fixed *ρ*_b_ (>*M*_halo_), or equivalently fixed peak height *ν* (right). The most extreme L23 galaxy candidates are shown as blue stars, with uncertainties indicating 68% intervals (symmetric about the median) of the posterior probability distribution. The existence of a galaxy with *M*_⋆_ at redshift *z* requires that such galaxies have a cumulative co-moving number density that is, at most, the number density shown in the left panel, as those galaxies must reside in host halo of mass *M*_halo_ = *M*_⋆_/(*f*_b_*ϵ*). The cumulative co-moving number density corresponding to an observed *M*_⋆_ will probably be (much) smaller than is indicated here, as the curves are placed on the plot by assuming the physically maximal *ϵ* = 1.0. For smaller values of *ϵ*, the curves in each panel move down relative to the points by a factor of *ϵ* (as indicated by the black downward-facing arrows). The right panel demonstrates that even for the most conservative assumption of *ϵ* = 1.0, the data points correspond to very rare peaks in the density field, implying a limited baryonic reservoir that is in tension with the measured stellar masses of the galaxies.

The left panel of Fig. 1 shows the relationship between the maximal inferred stellar mass for a given *M*_halo_, *M*_⋆_ = *f*_b_*M*_halo_ (that is, assuming the maximal *ϵ* = 1.0) and redshift *z* for fixed cumulative co-moving halo number densities ranging from 10^−10^ Mpc^−3^ (light grey) to 10^−2^ Mpc^−3^ (yellow). The curves evolve rapidly with redshift, with the maximal stellar mass corresponding to a fixed cumulative co-moving halo number density increasing by three orders of magnitude from *z* = 20 to *z* = 5. This rapid rise indicates that the mass reservoir available for the most massive galaxies increases quickly with redshift at fixed halo number density. The two most massive high-redshift galaxy candidates from the Labbé et al.^8^ (hereafter L23) sample, at *z* ≈ 7.5 (*M*_⋆_ ≈ 10^11^ *M*_⊙_) and *z* ≈ 9.1 (*M*_⋆_ ≈ 10^10.5^ *M*_⊙_), are shown as blue stars. These objects are unexpectedly massive, with stellar content reflective of haloes that have cumulative co-moving number densities no higher than ≈10^−5.2^ Mpc^−3^ (if *ϵ* = 1.0); for *ϵ* = 0.32 (0.10), the implied number density is ≈10^−7^ (10^−9.3^) Mpc^−3^. By comparison, the candidates were found in a survey of 38 arcmin^2^, a volume of *V* ≈ 10^5^ Mpc^3^ at each of the redshift bins—7 < *z* < 8.5 and 8.5 < *z* < 10—considered by L23.

The right panel of Fig. 1 recasts the issue in terms of the scarcity of systems as measured by cumulative mass density. In extended Press–Schechter models, the peak height *ν*(*M*_halo_, *z*) = *δ*_c_/*σ*(*M*_halo_, *z*) of an object—where *δ*_c_ ≈ 1.7 is the linear collapse threshold and *σ*^2^(*M*_halo_, *z*) is the variance of the linear density field at redshift *z* smoothed on a scale containing an average mass of *M*_halo_—is a measure of the fraction of mass in the Universe contained in virialized objects more massive than *M*_halo_ at redshift *z*. Typical haloes at *z* have *ν* = 1, which corresponds to 24% of the mass in the Universe residing in haloes at least that massive; larger values of *ν* indicate increasingly massive and therefore rare peaks in the density field at that epoch. The co-moving baryon density for each peak height in the figure is given in the legend; multiplying this number by the volume of a survey gives the total amount of baryons contained above the mass corresponding to that peak height and redshift. The L23 galaxies have peak heights of at least *ν* = 4.5 (assuming *ϵ* = 1.0), meaning that, at most, a fraction 6.2 × 10^−5^ of the baryons in the Universe are contained in haloes massive enough to host these galaxies. For reference, *ν* = 4.5 at *z* = 0 corresponds to *M*_halo_ ≈ 5 × 10^15^ *M*_⊙_. Adopting more reasonable efficiencies of *ϵ* = 0.32 or 0.10 results in rarer peaks with *ν* ≈ 5.4 or 6.4.

**Fig. 2:**
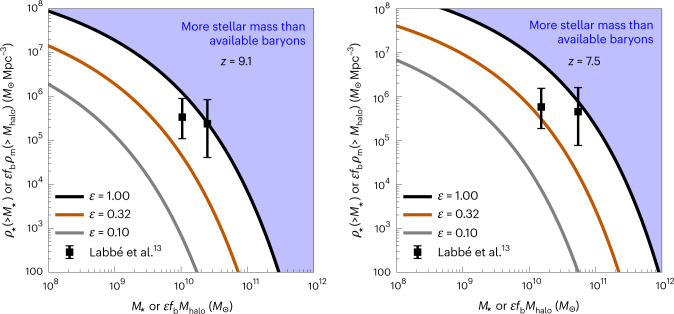
Stellar mass density limits. The co-moving stellar mass density contained within galaxies more massive than *M*_⋆_ at *z* ≈ 9.1 (left) and *z* ≈ 7.5 (right) for three values of the assumed conversion efficiency *ϵ* of a halo’s cosmic allotment of baryons into stars. Only if all available baryons in all haloes with enough baryons to form the galaxies reported by L23 have indeed been converted into stars by that point—an unrealistic limit—is it possible to produce the stellar mass density in the highest *M*_⋆_ bin at *z* ≈ 9 measured by L23 in a typical volume of a *Λ*CDM Universe with the Planck 2020 cosmology. Results are similar at *z* ≈ 7.5. For more realistic values of *ϵ*, the required baryon reservoir is substantially larger than the theoretical maximum in this cosmology. When considering 1 σ shot noise and sample variance errors added in quadrature (which comprise the uncertainties on the L23 data points in each panel), the measurements are consistent with the base *Λ*CDM model if *ϵ* > 0.57, which would still imply incredibly efficient star formation in the high-redshift Universe.

Figure 2 shows the cumulative stellar mass density reported by L23 at *z* ≈ 9 (left) and *z* ≈ 7.5 (right). The data, which come from individual massive objects, lie at the extreme of *Λ*CDM expectations, even in the most optimistic scenario: at both redshifts, the measurements lie at the theoretical limit of *ρ*_⋆_(>*M*_⋆_) = *f*_b_*ρ*_m_(>*M*_⋆_/*f*_b_), implying physically implausible values of *ϵ*(*z* ≈ 9) = 0.99 and *ϵ*(*z* ≈ 7.5) = 0.84. When considering the 1*σ* error (which incorporates uncertainties from Poisson fluctuations and sample variance added in quadrature), the data become marginally consistent with the available baryon reservoirs for an efficiency of *ϵ*(*z* ≈ 9) ≥ 0.57, which is probably an unrealistically high value. Assuming a more plausible value of *ϵ* = 0.10 or 0.32 yields a strong discrepancy with *Λ*CDM expectations at both redshifts, even when considering observational uncertainties.

## Discussion

The first glimpse of high-redshift galaxy formation with JWST has revealed surprisingly massive galaxy candidates at early cosmic times. These systems provide a way to test a bedrock property of the *Λ*CDM model (or alternately, assumptions in derivations of stellar masses or the viability of high-redshift galaxy candidates): the stellar content of haloes should not exceed the available baryonic material in those haloes. This requirement does not rely on assumptions such as abundance matching, but rather is simply a statement about the distribution of virialized mass in the Universe as a function of redshift and the baryonic reservoirs associated with those virialized haloes: galaxies of mass *M*_⋆_ can only form if haloes of mass *M*_⋆_/(*ϵf*_b_) have formed. It is also more stringent than the requirement that the observed galaxy ultraviolet luminosity function not exceed the theoretical maximum coming from a nearly instantaneous (10 Myr) conversion of a halo’s full baryonic reservoir into stars^15^, as it is an integral constraint as opposed to a differential one. The massive, high-redshift galaxy candidates catalogued in L23 lie near or at the stellar mass density constraint in *Λ*CDM.

There are several sources of observational uncertainty that enter these results. The flux calibration of NIRCam is continually being updated; L23 use calibrations that take into account updated detector offsets that are not yet part of the official JWST reduction pipeline (see, for example, Boyer et al.^16^ for examples of this effect and Nardiello et al.^17^ for related discussions of empirical point spread function modelling for JWST). With NIRCam photometry, a Balmer or 4,000 Å break at *z* ≈ 5 can be mistaken for a Lyman *α* break at *z* ≳ 12 (ref. ^18^); the L23 sample was selected to contain both Lyman and Balmer breaks, however, and is at low enough redshift (relative to *z* ≈ 15 sources) that NIRCam filters can typically exclude *z* ≈ 5 photometric solutions. The resulting photometric redshift estimates have single, narrow (*σ*_*z*_ ≈ 0.25) peaks. The masses of the galaxies are computed using the median of four methods for fitting the photometry (see L23 for details) and assume a Salpeter^19^ initial mass function. Different assumptions about the photometry (in particular, properties of nebular emission lines or the presence of an accreting supermassive black hole) or initial mass function could affect the derived stellar masses. The mass of the candidate at *z* ≈ 7.5 was also corrected for the possibility of amplification by mild gravitational lensing; this effect is estimated by L23 to be 0.15 dex, and the reported mass (and stellar mass density) of this object are therefore reduced by this amount to compensate. The error bars in Fig. 2 include errors in the volume estimates coming from both sample variance and Poisson noise, with the latter always being dominant in the regime considered here^1,20^.

The discrepancy between the observed high-redshift galaxy candidates and *Λ*CDM expectations is robust to uncertainties in cosmological parameters in the base *Λ*CDM model: the precision on each of the relevant parameters is at the ≲1% level^12^. Intriguingly, extensions to the base *Λ*CDM with enhanced values of *σ*_8_, *n*_s_ and the physical matter density *Ω*_m_*h*^2^—such as some early dark energy (EDE) models whose aim is to resolve the Hubble tension—predict earlier structure formation and a higher abundance of haloes at fixed mass at high redshift^21^, which would enhance the baryonic reservoirs available for forming early massive galaxies. Taking the best-fit EDE parameters from Smith et al.^22^, the cumulative co-moving baryonic density contained in galaxies more massive than *M*_⋆_=*f*_b_*M*_halo_ for the most massive L23 galaxy candidate at *z* ≈ 9.1 is a factor of 3.1 larger in EDE than in base *Λ*CDM, which is non-negligible; the L23 data points would then lie at *ϵ* = 0.74 instead of *ϵ* = 0.99. However, this EDE cosmology is in stronger tension with values of ${S}_{8}={\sigma }_{8}\,\sqrt{{\varOmega }_{{{{\rm{m}}}}}/0.3}$ measured at low redshift and predict that the Universe is ≈13 billion years old (as opposed to 13.8 billion years in the base *Λ*CDM model), which is in moderate tension with the measured ages of ultra-faint galaxies and globular clusters^23^.

At the redshifts studied here, *z* ≈ 7–10, the Sheth–Tormen mass function overestimates the abundance of massive haloes by 20–50% relative to numerical simulations^24–27^, meaning their true abundance at high redshift is probably lower than the Sheth–Tormen prediction and the constraints derived here are conservative. However, the lack of detailed comparisons between theory and simulations at high redshifts and high masses points to the importance of continued theoretical work in understanding the universality and applicability of halo mass function parameterizations in regimes relevant for JWST observations (and other forthcoming observatories).

The tension discussed in this paper is straightforward: the masses measured by L23 are only consistent with expectations from the standard cosmological model at the reported redshifts if star formation in the earliest phases of galaxy formation is incredibly efficient (*ϵ* ≥ 0.57). In the low-redshift Universe, such efficiencies are never seen, with *ϵ* ≲ 0.2 for all galaxies. The theoretical expectation is that efficiencies do indeed increase at high redshift^28^, although *ϵ* ≳ 0.57 is still highly extreme and probably implausibly high. If the explanation of the L23 galaxies is indeed a very high star formation efficiency, it implies that the star formation histories of such systems must rise steeply with time, following the behaviour of the baryon reservoirs inside virialized structures in *Λ*CDM. The results presented here could also be explained if the stellar initial mass function differs substantially from the assumed Salpeter form, the black hole accretion contributes significantly to the galaxies' emitted light or the volumes currently surveyed turn out to be highly atypical.

If none of these explanations holds up and these massive galaxies are spectroscopically confirmed, they will pose a serious challenge for *Λ*CDM structure formation with parameters given by Planck Collaboration^12^ because they signify the existence of a larger reservoir of collapsed baryons than is possible in this model. Forthcoming wider field JWST surveys, along with JWST spectroscopy of massive galaxy candidates, should be able to quickly confirm or refute the existence of this tension. Furthermore, the compatibility of any additional high-redshift galaxies or galaxy candidates discovered in JWST observations with *Λ*CDM expectations can be assessed in a straightforward way via Fig. 1. If analysis of JWST data continues to reveal the presence of strikingly massive galaxies at very early cosmic epochs, more exciting surprises lie ahead for the fields of galaxy formation and cosmology.

## Data Availability

Data from L23, including stellar mass estimates and photometric redshifts, are available at https://github.com/ivolabbe/red-massive-candidates; this paper uses data from sample_revision3_2207.12446.ecsv, commit 59fbbfa (from 2 January 2023).

## Code availability

All calculations that go into the figures in this paper are publicly available at https://github.com/mrbk/JWST_MstarDensity.
